# A New Strategy for As(V) Biosensing Based on the Inhibition of the Phosphatase Activity of the Arsenate Reductase from *Thermus thermophilus*

**DOI:** 10.3390/ijms23062942

**Published:** 2022-03-09

**Authors:** Rosanna Puopolo, Giovanni Gallo, Danila Limauro, Patrizia Contursi, Gabriella Fiorentino

**Affiliations:** 1 Department of Biology, University of Naples Federico II, Via Cinthia 21, 80126 Napoli, Italy; rosanna.puopolo@unina.it (R.P.); giovanni.gallo2@unina.it (G.G.); limauro@unina.it (D.L.); contursi@unina.it (P.C.); 2 Consiglio Nazionale delle Ricerche CNR, Institute of Polymers, Composites and Biomaterials (IPCB), Via Campi Flegrei, 34, Pozzuoli, 80078 Napoli, Italy

**Keywords:** arsenic, thermostable arsenate reductase, *Thermus thermophilus*, biosensor

## Abstract

Arsenic (As) pollution is a widespread problem worldwide. In recent years, biosensors based on enzymatic inhibition have been developed for arsenic detection, making the study of the effect of inhibitors on the selected enzymatic activity crucial for their setup. The arsenate reductase of *Thermus thermophilus* HB27, *Tt*ArsC, reduces As(V) into As(III), but is also endowed with phosphatase activity. This work investigates the inhibitory effects of As(V) and As(III) on phosphatase activity by taking advantage of a simple colorimetric assay; the results show that both of them are non-competitive inhibitors affecting the Vmax but not the K_M_ of the reaction. However, their Ki values are different from each other (15.2 ± 1.6 μM for As(V) and 394.4 ± 40.3 µm with As(III)), indicating a higher inhibitory effect by As(V). Moreover, the inhibition-based biosystem results to be selective for As(V) since several other metal ions and salts do not affect *Tt*ArsC phosphatase activity; it exhibits a sensitivity of 0.53 ± 0.03 mU/mg/μM and a limit of detection (LOD) of 0.28 ± 0.02 μM. The good sensitivity and specificity for As(V) point to consider inhibition of *Tt*ArsC phosphatase activity for the setup of a novel biosensor for the detection of As(V).

## 1. Introduction

Arsenic is a toxic metalloid, commonly occurring as a groundwater pollutant. It largely derives from mines, industrial wastes, or geochemical processes, and both its organic and inorganic forms can be found in the environment [1]. Among inorganic arsenic species, arsenate As(V) and arsenite As(III) are the most common and toxic forms, with As(III) being almost 60 times more toxic than As(V), the predominant form found in oxidized environment [2,3]. While As(V) toxicity is caused by its structural similarity with the phosphate ion, which determines the inhibition of oxidative phosphorylation, As(III) toxicity is mainly caused by its high affinity towards the thiol groups of proteins and cofactors, which determines their irreversible inhibition [4,5]. The identification of arsenic species is relevant to better understand their distribution, transformation in the environment, toxicity, metabolism, and health effects [6]. Indeed, arsenic is one of WHO’s 10 chemicals of major public health concern; millions of people all over the world are exposed to arsenic concentrations much higher than the guideline value (10 µg/L in drinking water), and the effects of long-term exposures at toxic concentrations are the cause of many diseases that, in extreme cases, lead to death [4]. For these reasons, in the 2030 Agenda for Sustainable Development, the indicator of “safely managed drinking water services” aims to guarantee all people access to drinking water free of microbial and chemical contaminants, including arsenic, which must be hence monitored [3,7,8,9]. The traditional approaches for monitoring arsenic pollution are based on chemical or physical analysis and allow for highly accurate and sensitive determination of the exact composition of any sample. These analyses require specialized operators and expensive instruments, such as the ICP-MS (Inductively Coupled Plasma-Mass Spectrometry), which, to date, is the most adopted technique for detecting heavy metals [10]. Furthermore, if it is desired to obtain information on arsenic species present in the samples to be analyzed, ICP-MS methodologies need to be implemented with other techniques, such as high-performance liquid chromatography (HPLC) [11]. To date, the efficient separation of arsenic compounds is a critical step in speciation analysis, either for routine analysis of known arsenic compounds or for the identification of unknown compounds [6]. The need for accurate and less expensive measurements has led to the development of biosensors based on biomolecules. These analytical devices integrate a biological recognition element with a physical transducer to generate a measurable signal proportional to the concentration of the analyte [12]. Thanks to the biological component, biosensors provide a more eco-sustainable alternative to toxicity measurements than the classical approaches [12]. In recent years, many biosensors for arsenic detection have been developed in which the biocomponent is a whole-cell or a biomolecule such as an aptamer, DNA, or an enzyme [13]. However, despite demonstrations of proof-of-principles, the successful application of arsenic biosensors on environmental samples has been limited by the low sensitivity, specificity, and/or stability of the biological component [14,15]. Whole-cell biosensors have the advantages of specificity, low cost, ease of use, portability, and continuous real-time signal emissions, but their use is limited by the necessity to have long incubation periods and high detection limits [16,17,18]. On the other hand, cell-free biosensors have faster response times but may still lack stability and/or specificity [17,19]. To overcome the drawback of enzyme stability, many studies have been focused on the realization of more stable enzymes obtained through protein engineering [20,21], as well as the use of novel surface nanostructured biosystems [22], or enzymes immobilized on nanomaterials [23,24]. Attention has also been devoted to the employment of extremozymes as sources of stable biomolecules [25,26,27]. For example, thermozymes from thermophilic microorganisms are active at high temperatures and in several harsh conditions, such as high concentrations of heavy metals, salts, and low pH [27,28,29,30,31,32,33]. In recent times, the development of arsenic biosensors that measure arsenic through enzymatic inhibition has been described. Inhibition-based biosensors quantify the analyte by measuring the proportional reduction of the selected enzymatic activity and are considered very useful as indicators of general toxicity for the fast identification of contaminated samples [34]. To date, inhibition biosensors have been of interest in the field of clinical biochemistry to quantify enzymes of specific biological pathways specifically inhibited by drugs or to detect toxic compounds in food and environmental samples [35,36]. For example, Sanllorente and co-workers developed electrochemical biosensors for both As(III) and As(V), the first was based on the inhibition of an acetylcholinesterase and the latter on that of an acid phosphatase [37,38]. These biosensors have detection limits in the range of permissible exposure limits for arsenic, but their application is compromised by the low specificity caused by the presence of other ions in the samples [17,19]. 

The arsenate reductase from the thermophilic bacterium *Thermus thermophilus* HB27, *Tt*ArsC [39], is a thermostable enzyme exploited in the setup of arsenic biosensors thanks to its ability to react with As(V) [40,41]. *Tt*ArsC was discovered as a component of the arsenic resistance system of *T. thermophilus* HB27 that catalyses the reduction of As(V) to As(III) [39]. The more toxic As(III) is then actively extruded by a membrane P_1B_ type ATPase, named *Tt*ArsX [42], or methylated by a recently identified arsenate reductase, *Tt*ArsM [43]. These genes are regulated at the transcriptional level by the metal responsive transcriptional repressor, *Tt*SmtB [44,45]. *Tt*ArsC belongs to the subfamily of thioredoxin-coupled arsenate reductases, whose most known enzyme is ArsC from the *Staphylococcus aureus* plasmid pI258 [46]. Members of this family are characterized by the presence of three redox-active cysteines, one (Cys7 in *Tt*ArsC) performing the nucleophilic attack to As(V), and the other two (Cys 83 and Cys 90) forming a disulphide bond after substrate reduction [46]. To regenerate the enzyme, reducing equivalents flow from NADPH to thioredoxin reductase to thioredoxin, reducing the formed Cys-Cys disulphide bond [46]. In members of this subfamily, the N-terminal catalytic cysteine is encompassed in a conserved CysX_5_Arg motif, known as the P-loop homologous to that found in low molecular weight phosphotyrosine phosphatases; indeed, many thioredoxin-coupled arsenate reductases, including *Tt*ArsC, are endowed with phosphatase activity [47]. From an evolutionary point of view, it has been proposed that arsenate reductase activity could have evolved from phosphatase activity through a change in mechanism [46,47].

In this work, we investigate the inhibitory effect of arsenic on *Tt*ArsC phosphatase activity to evaluate its exploitation as an optical biosensor. 

## 2. Results and Discussion

Recombinant *Tt*ArsC was produced and purified to homogeneity from *E. coli* cells as already reported [39]. As shown, *Tt*ArsC possesses a secondary phosphatase activity, which can be easily measured with a colorimetric assay following the release of p-nitrophenol at 60 °C [20]. Indeed, detection of phosphatase activity does not need the intervention of the redox cascade system composed of NADPH, thioredoxin reductase, and thioredoxin, which is required to restore the reduced state of the two catalytic cysteines in the enzyme [39]. In this paper, to verify the potential employment of the enzyme as an optical biosensor to detect arsenic, we investigated the effects of arsenic and other metal ions on *Tt*ArsC phosphatase activity. 

### 2.1. Arsenic Inhibition Profile

**Figure 1:**
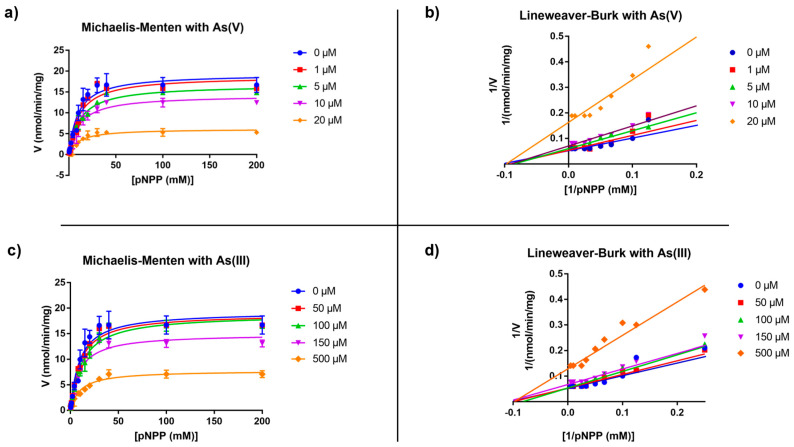
Graphical representation of Michaelis–Menten kinetics at different concentrations of (**a**) As(V) and (**c**) A(III) and their relatives Lineweaver–Burk double reciprocal plots in presence of (**b**) As(V) and (**d**) As(III).

The *Tt*ArsC phosphatase activity was measured spectrophotometrically to determine the kinetic parameters. In particular, we developed Michaelis–Menten and Lineweaver–Burk double-reciprocal plots, which indicated for *Tt*ArsC a k_cat_ of 0.005 s^−1^ and a K_M_ of 9.57 ± 1.95 mM, with an R^2^ value of 0.9571. Then, the inhibitory effects of As(V) and As(III) were evaluated as described below. Figure 1 shows the effect of increasing concentrations of As(V) (panel a, b) and As(III) (panel c, d) on the corresponding plots, while the R^2^ values for individual Michaelis–Menten fittings are reported in Table 1 for As(V) and Table 2 for As(III).

#### 2.1.1. As(V) Inhibition 

The increase in As(V) concentration, from 0 to 20 µM, resulted in a decrease in V_max_ from approximately 19.29 ± 1.14 to 6.14 ± 0.33 nmol/min/mg (Figure 1a, Table 1). On the other hand, the K_M_ constant resulted in almost unvaried, indicating a mechanism of non-competitive inhibition. As fact, the data fit to a non-competitive inhibition model elaborated through a nonlinear regression, which yielded an overall R^2^ value of 0.9499 [48,49]. Accordingly, Lineweaver–Burk double-reciprocal plots also indicated a non-competitive inhibition by As(V). 

#### 2.1.2. As(III) Inhibition 

Similarly, a decrease in V_max_ and an almost unvaried K_M_ were observed at increasing As(III) concentrations. The V_max_ values changed from approximately 19.29 ± 1.14 to 7.79 ± 0.43 nmol/min/mg; however, the inhibitory effect was obtained at a As(III) concentration much higher than As(V) (Figure 1c and Table 2). Also in this case, the Lineweaver–Burk double-reciprocal plot indicated a non-competitive inhibition, confirmed by data fitting to the non-competitive inhibition model with an R^2^ value of 0.9633.

According to models of non-competitive inhibition for both As(V) and As(III), the Ki values determined were 15.2 ± 1.6 µm for As(V) and 394.4 ± 40.3 μM for As(III), showing that the affinity of *Tt*ArsC towards As(V) is almost 25 times greater than that towards As(III); such a difference could be due to the fact that As(V) is the substrate of *Tt*ArsC reductase activity. In *Tt*ArsC, the catalytic nucleophile Cys7 is also the first amino acid of the P-loop; therefore, it could be involved in both phosphate and arsenate binding, while the other two conserved Cys residues (Cys82 and Cys89), essential for the reduction of As(V) into As(III), are spatially separated from the P-loop [39] (Supplementary Figure S1). In fact, through NMR analysis, Messens and co-workers demonstrated that both arsenate and phosphate ions bind the first Cys residue of the P-loop of ArsC from *S. aureus* pI258 [47].

### 2.2. As(V) Dose-Response Inhibition

**Figure 2:**
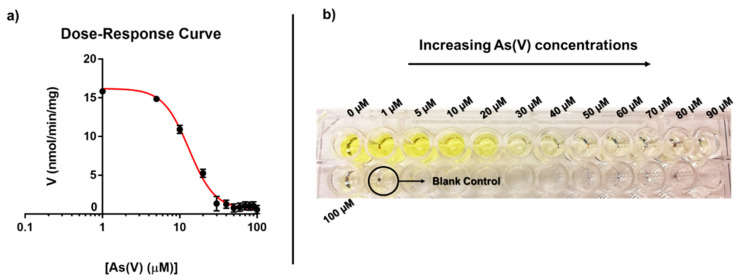
(**a**) Dose-response curve of the inhibition of *Tt*ArsC phosphatase activity at increasing As(V) concentration was (reported in panel b). (**b**) *Tt*ArsC phosphatase activity assay in a multiwell plate at increasing concentrations of As(V).

Considering that the Ki value towards arsenate indicates that *Tt*ArsC activity is inhibited by the As(V) concentration in a micromolar range, we decided to obtain an As(V) dose-response curve to derive an equation for calculating the unknown concentration of As(V) in solution. Activity assays were performed using 40 mM of pNPP and varying As(V) concentrations. The values of *Tt*ArsC specific activity in the function of As(V) concentration were fitted to the following model: [Inhibitor] vs response [50] (Figure 2a). This model allows determining the IC50 of the inhibitor, i.e., the concentration that provokes a response halfway between the maximal (T) activity in the absence of the inhibitor and the maximally inhibited (B) activity. Figure 2a shows that increasing concentrations of As(V) determine an inhibition profile described by the following Equation (1):

(1) $$y=B+\frac{\left(T-B\right)}{1+\frac{x^{Hill\;Slope}}{IC50^{Hill\;Slope}}}$$

where T and B are 16.20 ± 0.32 and 0.59 ± 0.24 nmol/min/mg, respectively; IC50 is 13.16 ± 0.63 µm and the Hill slope, is 2.60 ± 0.26. Since this value is higher than that of a dose-response curve with a standard slope (1.0), it can be argued that As(V) is a strong inhibitor. Indeed, the more inhibitory is a particular substance, the steeper will be the curve [50,51]. The R^2^ value was = 0.9959, indicating that the data fit well with the regression model.

Figure 2b shows that at As(V) concentrations higher than 50 µM, enzyme activity is completely inhibited since the solution appears colourless. Therefore, with this As(V) concentration, it is possible to evaluate the inhibition with the naked eye. We also determined the LOD according to the 3SD/m criterion, where m is the sensitivity, i.e., the slope of the dose-response in its linear range, and SD is the standard deviation of the blank [52,53]. In our case, the linear range was considered between 5 and 30 µM, and the blank SD value was 0.05 mU/mg/μM. The inhibition system exhibited a sensitivity of 0.53 ± 0.03 mU/mg/μM and a LOD of 0.28 ± 0.02 μM. Other characterized As(V) inhibition-based biosensors displayed similar performances [13,17]; for example, the amperometric AcP biosensor tested on groundwater samples presented a LOD of 0.11 μM in a linear range of 0.1 to 1.3 μM of As(V) [38]; the self-powered laccase biosensor showed a LOD of 132 μM and sensitivity 0.98 ± 0.02 mV/mM [54,55] and the acid phosphatase-polyphenol oxidase biosensor had a LOD of 2 nM in a As(V) linear range of 8.9 to 79 nM [56]; these two were also tested to measure As(III); the first exhibit LOD of 13 µM and sensitivity of 0.91 ± 0.07 mV/mM [54,55], while the latter is more specific since does not suffer interferences by As(III) [56]. 

### 2.3. Other Metals

**Figure 3:**
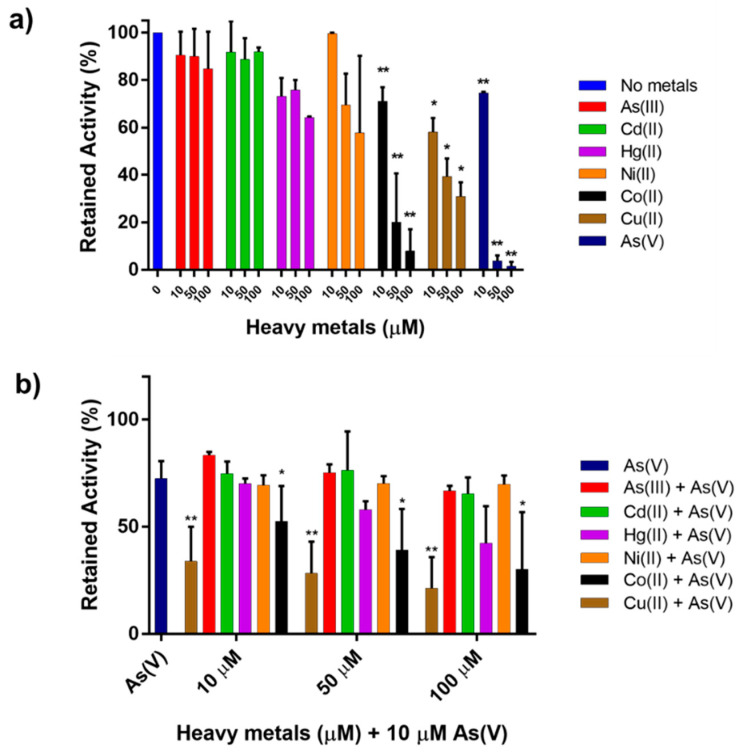
(**a**) Retained activity of *Tt*ArsC in presence of 10, 50, and 100 μM of heavy metals; (**b**) Retained activity of *Tt*ArsC in presence of a fixed concentration of As(V) (10 μM) and increasing concentrations of other heavy metals (10, 50, and 100 μM). Heavy metals are reported as follows: As(V) in dark blue, As(III) in red, Cd(II) in green, Hg(II) in purple, Ni(II) in orange, Co(II) in black, and Cu(II) in brown. Statistical analysis was performed through the ordinary one-way ANOVA on GraphPad Prism 7.00; significant differences with respect to (**a**) *Tt*ArsC activity without metals and (**b**) to *Tt*ArsC activity in presence of As(V) (10 μM) are indicated as follows: * *p* < 0.05.

In order to exploit the possibility of turning the inhibition of phosphatase activity into a biosystem for As(V) detection in water matrices, we evaluated the interference of As(III) and other metal ions on *Tt*ArsC inhibition by As(V) [57]. In particular, the residual phosphatase activity was measured after addition to the enzymatic mix of As(III), Cd(II), Hg(II), Ni(II), Co(II), or Cu(II), at three different concentrations (10, 50, or 100 μM), considering as 100% the activity in the absence of metals. The inhibitory effect of the metal ions was compared with that of As(V) and analyzed either in the absence or in the presence of the latter. As shown in Figure 3a, a significant inhibition of phosphatase activity was observed only in the presence of Co(II) and Cu(II); on the other hand, the activity measured in the presence of As(III), Cd(II), Hg(II), and Ni(II) was similar to the control without metals, suggesting that these metals were not inhibitory and did not influence As(V) detection. Since no effect on activity was observed with the majority of metal ions tested, we asked whether the specific inhibitory effect of As(V) was affected by the co-presence of heavy metals. Therefore, the phosphatase activity was tested at a fixed concentration of As(V) (10 µM) close to the IC50 (see above), adding the other metal ions separately (Figure 3b). As can be seen from the figure, except for Co(II) and Cu(II), the presence of As(III), Cd(II), Hg(II), and Ni(II) did not alter to a significant extent the inhibitory effect of 10 μM As(V); indeed, they reduced phosphatase activity to ~75%, which by the addition of Co(II) and Cu(II) decreased to ~30% and ~20%.

Altogether, these results highlight the applicability of the system towards the detection of As(V) in the presence of As(III) Cd(II), Hg(II), and Ni(II); this result is of interest considering that many biosensors for heavy metal detection are based on the inhibition of phosphatase activity [55,56].

Moreover, in the context of arsenic speciation, the inhibition of phosphatase activity would discriminate between As(V) and As(III) at least in a range from 10 to 100 μM [3,6].

### 2.4. Aqueous Saline Solutions

**Figure 4:**
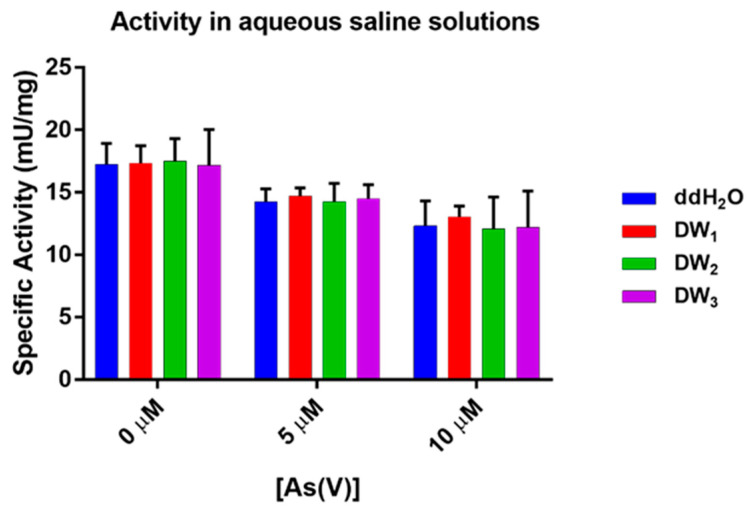
*Tt*ArsC phosphatase activity performed in ddH_2_O, three drinkable waters (DW), and in the presence of As(V) 5 and 10 μM.

Since the data obtained with the above-considered metal ions have shown that most of them do not interfere with the inhibition of *Tt*ArsC phosphatase activity by As(V), we wondered if more complex solutions in which more metals are present at the same time could have effects. Since drinking waters contain magnesium, calcium, sodium, potassium salts, bicarbonates, sulfates, nitrates, and nitrites in different concentrations, we decided to measure *Tt*ArsC phosphatase activity by incubating the enzyme in three different commercial drinking waters and evaluating if inhibition occurred in comparison to an enzyme assay performed in canonical buffer. The results indicate that in the presence of these more complex solutions, *Tt*ArsC retains its specific phosphatase activity. Therefore, in order to assess whether under these conditions *Tt*ArsC was still inhibited by As(V), we performed the same experiments also in the presence of 5 and 10 μM As(V); the results shown in Figure 4 highlight that no significant differences occurred when comparing the reactions performed in ddH_2_O with the corresponding performed in mineral water, suggesting that the inhibitory dose-response effect of As(V) on *Tt*ArsC is specific. These results encourage to exploit the system on other water samples.

## 3. Materials and Methods

### 3.1. Chemicals

The metal salts used in this work were purchased by Sigma-Aldrich and are the following: sodium (meta) arsenite (NaAsO_2_); sodium arsenate dibasic heptahydrate (Na_2_HAsO4 · 7H2O); cadmium chloride (CdCl_2_); cobalt chloride (CoCl_3_); copper chloride (CuCl_2_); mercury chloride, (HgCl_2_); nickel chloride (NiCl_2_). Also, the substrate 4-nitrophenyl phosphate disodium salt hexahydrate, pNPP, (CAS Number 333338-18-4) was purchased from Sigma Aldrich. 

### 3.2. Heterologous Expression and Purification of TtArsC from E. coli

Recombinant *Tt*ArsC was purified to homogeneity from BL21-CodonPlus(DE3)-RIL cells transformed with pET30/*TtarsC* vector using the procedure already described, consisting of a thermo-precipitation of the *E. coli* cell extract followed by anion exchange (Resource Q, GE Healthcare) and gel filtration (Superdex S75 GE Healthcare) chromatographies. To prevent protease activity and cysteine oxidation, an inhibitor cocktail (Roche) and 1 mM DTT were respectively added to all the buffers, and the solutions were flushed with nitrogen prior to use [39]. 

### 3.3. Phosphatase Activity Assay

The phosphatase activity of purified *Tt*ArsC was measured at 60 °C using pNPP as substrate (Sigma-Aldrich) and following the increase in absorption at 405 nm in continuous for 1 h, due to the formation of p-nitrophenol (ε_405_ = 18,000 M^−1^ cm^−1^). Each reaction was performed in triplicate in a plate reader spectrophotometer (Sinergy H4, software Version 2.07.17) in a total volume of 160 μL containing 4 μM *Tt*ArsC in 20 mM Tris-HCl pH 7. Substrate concentrations ranged from 0.25 to 200 mM (see below). As a negative control, the same reactions were performed without the enzyme (blank control). The variation of A_405nm_ per min (ΔOD/min) obtained in the absence of enzymes was subtracted from that obtained from the experiments with enzymes. One unit of enzyme activity (U) was defined as the amount of enzyme required to release 1 μMol of p-nitrophenol per min under the described assay conditions; therefore, 1 nmol/min/mg corresponds to 1 mU/mg.

### 3.4. Inhibition Assays 

The effect of As(V) and As(III) on phosphatase activity was determined by measuring the phosphatase activity with 4 μM *Tt*ArsC in 20 mM Tris-HCl pH 7, in the presence of fixed concentrations of metal ions at varying pNPP substrate concentrations. Michaelis–Menten curves were obtained measuring the enzyme activity in the presence of 0–1–5–10–20 μM of As(V) or 0–50–100–150–500 μM of As(III). For each curve determination, the pNPP substrate concentration varied from 0.25 to 200 mM. All the experimental sets were performed in triplicate.

### 3.5. As(V) Dose-Response Curve

The As(V) dose-response curve was obtained measuring the enzyme activity with saturating concentration of pNPP (40 mM) and As(V) at final concentration ranging from 0 to 100 μM. As a negative control, the same reaction was performed without enzyme and inhibitor (blank control). The experiments were performed in triplicate.

### 3.6. Metals Inhibition

The phosphatase activity of *Tt*ArsC in the presence of other heavy metal ions was also investigated. Cd(II), Hg(II), Ni(II), Co(II), and Cu(II) 10, 50, or 100 μM were added to reactions performed at saturating concentration of pNPP (40 mM); the residual activity was evaluated in comparison to the activity without metals, considered as 100%. The experiments were performed in triplicate. Moreover, to evaluate whether the metals affected the determination of the As(V) concentration, As(III), Cd(II), Hg(II), Ni(II), Co(II), and Cu(II) were added to reactions at final concentrations of 10–50-100 μM in the presence of a fixed As(V) concentration (10 μM). Experiments were performed using 40 mM pNPP. The percentage of retained activity in the presence of metals was evaluated in comparison to the activity without metals, considered as 100%. The experiments were performed in triplicate.

### 3.7. Complex Saline Solution Inhibition

*Tt*ArsC phosphatase activity assay was also tested in complex saline solutions, substituting the volume of ddH_2_O in the reaction (~130 µL) with commercial drinking water. Experiments were performed with a fixed concentration of pNPP (40 mM). To evaluate the interference of commercial waters on As(V) detection, As(V) was added in solutions at final concentrations of 5 and 10 μM. The salt composition of drinking water considered is that shown on the labels of commercial water and is reported in Table 3.

### 3.8. TtArsC 3D Modelling

The 3D structure model of *Tt*ArsC was elaborated using I-TASSER (https://zhanglab.ccmb.med.umich.edu/I-TASSER/, accessed on 6 March 2022), with an ab-initio approach. The model obtained shows a confidence score (C-score) of −0.81. After the model was visualized the cysteine residues were highlighted with colours using PyMOL v0.99 (https://pymol.org, accessed on 6 March 2022). 

### 3.9. Data Analysis

All the experiments were performed in triplicates. Data were processed using GraphPad Prism 7.00 to determine the kinetic parameters [50]; statistical analyses were performed through the ordinary one-way ANOVA; significant differences in *Tt*ArsC phosphatase activities are indicated as follows: * *p* < 0.05, ** *p* < 0.01, *** *p* < 0.001, **** *p* < 0.0001.

## 4. Conclusions

This work describes the set-up of a novel spectrophotometric detection system for As(V) in a solution based on the inhibition of the phosphatase activity of *Tt*ArsC. In our opinion, it represents a new interesting avenue in the development of arsenic biosensors, as the system might represent a new sustainable tool for arsenic speciation. One of the main advantages is represented by the fact that this method is quite easy and very feasible on a lab scale. In particular, the system exhibits a sensitivity of 0.53 ± 0.03 mU/mg/μM and a LOD of 0.28 ± 0.02 μM. Although the limits of detection are higher than those reported in limit guidelines, the strategy is simple enough to be applied in the preliminary investigations on waters in which the main contaminant is As(V) [4]. In fact, it is possible to see the complete inhibition of the reaction with the naked eye from 50 μM As(V) onwards, as in this range of As(V) concentration, a complete inhibition of the phosphatase activity is achieved.) Furthermore, all the other heavy metals that we tested (except for Co(II) and Cu(II)) caused enzyme inhibition only at higher concentrations, indicating that the detection capability of As(V) by *Tt*ArsC is not affected by the presence of such ions and/or molecules. In addition, the enzyme is effective in detecting As(V) in commercial waters. Improvements in sensitivity and specificity that might be achieved through protein engineering could have a profound impact on the practical application of the arsenic biosensor. In line with this principle, different mutants could be obtained and applied to measure arsenic on the basis of the kind of water sample that one wants to analyse. This strategy could lead to significant development in the field.

## Figures and Tables

**Table 1:** Kinetics parameters, V_max_ and K_M_, of the *Tt*ArsC phosphatase activity and respective R^2^ values for nonlinear regression fitting at different As(V) concentrations.

As(V) (µM)	V_max_ (nmol/min/mg)	K_M_ (mM)	R^2^
0	19.29 ± 1.14	9.57 ± 1.95	0.9571
1	18.77 ± 1.36	11.00 ± 2.65	0.9429
5	16.70 ± 0.64	11.75 ± 1.46	0.9827
10	14.22 ± 0.58	11.18 ± 1.52	0.9807
20	6.14 ± 0.33	10.27 ± 1.86	0.9660

**Table 2:** Kinetics parameters, V_max_ and K_M_, of the *Tt*ArsC phosphatase activity and respective R^2^ values for nonlinear regression fitting at different As(III) concentrations.

As(III) (µM)	V_max_ (nmol/min/mg)	K_M_ (mM)	R^2^
0	19.29 ± 1.14	9.57 ± 1.95	0.9571
50	18.91 ± 0.74	10.13 ± 1.34	0.9810
100	18.79 ± 0.77	12.24 ± 1.61	0.9811
150	14.95 ± 0.65	9.11 ± 1.39	0.9753
500	7.79 ± 0.43	10.20 ± 1.89	0.9552

**Table 3:** Salts composition reported in drinkable water (DW). All the values are considered as mg per liter of water. N.R. = Not Reported.

Salts		DW1 (mg/L)	DW2 (mg/L)	DW3 (mg/L)
Bicarbonate	HCO_3_^−^	321	215.0	498
Calcium	Ca^2+^	87.0	71.0	124
Magnesium	Mg^2+^	17.6	5.5	29.8
Silica	SiO_2_	8.0	16.6	N.R.
Nitrate	NO_3_	3	9.4	2
Sodium	Na^+^	4.9	11.7	4.0
Sulphates	SO_4_^2−^	25.6	10.7	17.6
Chlorides	Cl^−^	7.7	17.9	6.6
Potassium	K^+^	1.2	1.2	1.2
Fluorides	F^−^	<0.2	<0.10	N.R.
Ammonium	NH_4_^+^	N.R.	<0.05	N.R.
Nitrites	NO_2_^−^	N.R.	<0.002	<0.002

## Supplementary Materials

The following supporting information can be downloaded at: https://www.mdpi.com/article/10.3390/ijms23062942/s1.
