# Supplementary material for: A New Strategy for As(V) Biosensing Based on the Inhibition of the Phosphatase Activity of the Arsenate Reductase from Thermus thermophilus
Source: Int J Mol Sci. 2022 Mar 9;23(6):2942. doi: 10.3390/ijms23062942 (PMC8949286; doi:10.3390/ijms23062942)
Supplement: Supplementary file 1 [file ijms-23-02942-s001.zip › ijms-1537219-supplementary.pdf]

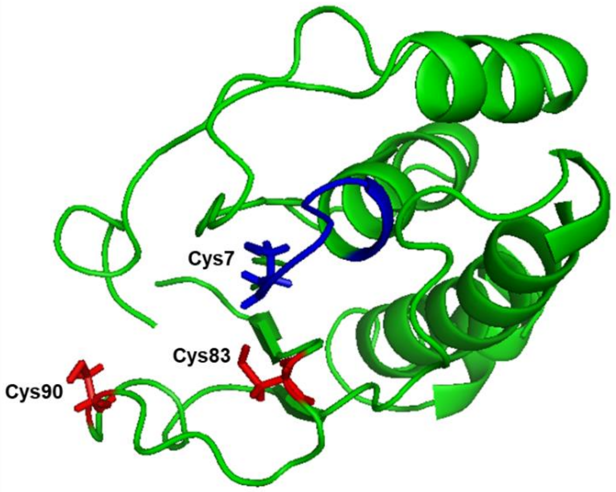

Figure S1: 3D model of *Tt*ArsC. The P-loop including the Cys7 residue is reported in blue; the other two Cys residues (Cys83 and Cys90) are coloured in red.
